# Author Correction: Confinement-induced accumulation and de-mixing of microscopic active-passive mixtures

**DOI:** 10.1038/s41467-022-33600-6

**Published:** 2022-10-03

**Authors:** Stephen Williams, Raphaël Jeanneret, Idan Tuval, Marco Polin

**Affiliations:** 1 Department of Physics, University of Warwick, Coventry, CV4 7AL UK; 2 Laboratoire de Physique de l’Ecole Normale Supérieure, ENS, Université PSL, CNRS, Sorbonne Université, Université de Paris, F-75005 Paris, France; 3 Departament de Física, Universitat de les Illes Balears, 07071 Palma de Mallorca, Spain; 4 Instituto Mediterráneo de Estudios Avanzados, IMEDEA, Miquel Marques 21, 07190 Esporles, Spain

**Keywords:** Biological physics, Statistical physics

**Correction to**: *Nature Communications* 10.1038/s41467-022-32520-9, published online 15 August 2022

In this article the funding from MCIN/AEI/ 10.13039/501100011033 was omitted. The original article has been corrected.

