# Supplementary material for: Confinement-induced accumulation and de-mixing of microscopic active-passive mixtures
Source: Nat Commun. 2022 Aug 15;13:4776. doi: 10.1038/s41467-022-32520-9 (PMC9378696; doi:10.1038/s41467-022-32520-9)
Supplement: Supplementary file 1 — Supplementary Information [file 41467_2022_32520_MOESM1_ESM.pdf]

Supplementary Materials: Confinement-induced accumulation and spontaneous de-mixing in a dilute colloidal active-passive mixture

Stephen Williams[1], Raphaël Jeanneret[2], Idan Tuval[3,4], Marco Polin[1,3,4,*]
[1] Department of Physics, University of Warwick, Coventry, CV4 7AL, United Kingdom
[2] Laboratoire de Physique de l'Ecole Normale Supérieure, ENS, Université PSL, CNRS, Sorbonne Université, Université de Paris, F-75005 Paris, France
[3] Departament de Física, Universitat de les Illes Balears, 07071 Palma de Mallorca, Spain
[4] Instituto Mediterráneo de Estudios Avanzados, IMEDEA, 07190 Esporles, Illes Balears, Spain*

SUPPLEMENTARY METHODS

1. Jump detection and effective diffusivity in the no-jump part of the dynamics

In this section we outline how the jumps are extracted from the trajectories of the colloids and how the parts of the trajectories that do not pertain to jumps are then used to estimate the effective diffusivity in the no-jump part of the dynamics.

In order to recognise the jump events, we use a method which is similar to both [1, 2], but with slight modifications. The process follows first the method of [1], using directional correlation between subsequent particle displacements to identify candidate periods of non-Brownian displacement. Subsequently, as in [2], the individual displacements during these periods are evaluated to ensure that the steps are sufficiently large, further confirming them as non-Brownian in origin.

To look for the directionally correlated windows we start by defining the displacements $\Delta\mathbf{r}(t) = \mathbf{r}(t+\Delta t) - \mathbf{r}(t)$, where $\mathbf{r}(t)$ is the colloid position at time t. With these we calculate the scalar product $p_{\Delta t}(t) = \Delta\mathbf{r}(t+\Delta t)\cdot\Delta\mathbf{r}(t)$. In the Brownian case $\langle p_{\Delta t}(t)\rangle = 0$ with a standard deviation given by $q_c = \sqrt{8}D_0\Delta t$. For a diffusivity $D_0 = 0.0439\,\mu\text{m}^2/\text{s}$, the thermal diffusivity for a $5\,\mu$m radius at 300 K in bulk water, and a time between frames $\Delta t = 0.1\,$s, we have $q_c = 0.0124\,\mu\text{m}^2$. Notice that, due to hydrodynamic interactions between colloids and boundaries, D_0 over-estimates the thermal diffusivity of the colloids in our experiments. This makes the threshold for classification of active jumps more stringent. From this we compute $q_{\Delta t}(t) = (p_{\Delta t}(t+\Delta t) + p_{\Delta t}(t))/2$, and use $4q_c$ as a threshold to select sections of a colloids' trajectory which are sufficiently directionally correlated, and could therefore be active jumps. Finally, we confirm that each of these potential jumps (consecutive time steps where $q_{\Delta t}(t)$ is above the threshold) has individual displacements which are sufficiently large to rule out correlated Brownian motions. To do this, we calculate for these trajectories the displacement magnitudes $|\Delta\mathbf{r}(t)| = |\mathbf{r}(t+\Delta t) - \mathbf{r}(t)|$, we can then compare these values to the Brownian expectation $\sqrt{4D_0\Delta t} = 0.132\,\mu$m. For a candidate jump trajectory, we require that at least 70% of the individual displacements within it are larger than the Brownian prediction for the overall jump to be confirmed. This percentage strikes a balance between being overselective -losing a large portion of actual jumps- and underselective -mis-classifying non-jump events as jumps. We have thoroughly tested this selection procedure by checking on the experimental movies that the different steps in the algorithm indeed reject non-jump events (~ 100 events checked, randomly selected), while selecting events arising from the interaction with nearby algae (~ 100 events checked, randomly selected).

Once the jump sections of the trajectories have been recognised, it is then possible to estimate D_{WJ}, the diffusivity that the colloids would have in absence of jumps. This returns $D_{WJ} \simeq 0.162 \pm 0.0057\,\mu\text{m}^2/\text{s}$. As expected, this is larger than the thermal contribution as the active displacements are not all jumps (see [2, 3]).

2. Poissonian approximation of the waiting time distributions

Using the digitised trajectories, after the jump sections have been identified, it is possible to identify the distribution of encounter rates as a function of the position of the colloids. To do this, for each individual colloidal trajectory,

* Correspondence: mpolin@imedea.uib-csic.es

we can look at the time elapsed between the nth and the $n+1$th jump. The position associated to the wait is then the end position of the nth jump y, since the diffusive motion during the waits in minimal. Using all the colloid's trajectories we can get reasonable statistics for the different positions throughout the channel, allowing us to construct the distribution of wait times $P(\tau, y)$.

Figure 2a in the main manuscript shows a semilog plot of the waiting time distributions for the $W = 100\,\mu$m case, for 10 uniformly spaced bins within the range of y-values that goes from the channel centre to the closest approach of the colloids to the side walls. The distributions for the other values of W are equivalent. All distributions share the same features. They are well described by a single exponential (straight line in semilog) beyond $\approx 3\,$s, but extrapolating this exponential to shorter times underestimates the actual number of events. As can be seen in the Supplementary Movie M3, the fundamental reason for this deviation is that, in some instances, after the initial interaction between an alga and a colloid, the colloid can alter the motility of the cell in a way that leads it to turn and interact again with the colloid. This generates an excess of interactions at short time, over those that would be estimated by a simple Poissonian process. Still, the process is only weakly non-Poissonian and for modelling purposes we will still describe the waiting time dynamics within each bin in terms of a single effective y-dependent rate $\lambda(y)$ and a distribution of active jumps that is independent of the waiting time elapsed. However, we need to find a reasonable way to extract these rates from the waiting time distribution. To do that, we start by considering the colloidal trajectories within a $120\,\mu$m-wide strip across the central position y_{ctr} of the $2W = 200\,\mu$m-wide channel. In this area the effective motion of the colloids is diffusive with a spatially constant diffusivity which we measure experimentally to be $D_{\text{eff}}^{\text{bulk}} = 1.82 \pm 0.02\,\mu\text{m}^2/\text{s}$. We assume that, besides thermal diffusion, the colloids' motion can be described in terms of active isotropic jumps of magnitude sampled independently from $P(\ell, y_{ctr})$ (see Supplementary Figure 4a) with a Poissonian waiting time dynamics with rate $\lambda(y_{ctr})$. In this central region, where λ and $\langle \ell^2 \rangle$ do not depend on space, the effective long-time diffusivity can then be written as $D_{\text{eff}}^{\text{bulk}} = D_{WJ} + \frac{1}{2}\lambda(y_{ctr})\langle \ell(y_{ctr})^2 \rangle$. Considering $D_{WJ} \simeq 0.162\,\mu\text{m}^2/\text{s}$ and the experimental distribution of jump magnitudes, giving $\langle \ell^2(y_{ctr}) \rangle \simeq 15.5\,\mu\text{m}^2$, the measured value of D_{eff} can be used to estimate the value of $\lambda(y_{ctr})$. We obtain $\lambda(y_{ctr}) \simeq 0.23\,\text{s}^{-1}$. This value should be compared with $\lambda(y_{ctr})_{lin} = 0.97 \pm 0.02\,\text{s}^{-1}$ obtained from the standard least squares fitting of the waiting time distribution, and with $\lambda(y_{ctr})_{log} = 0.32 \pm 0.01\,\text{s}^{-1}$ which instead is obtained by least squares fitting of the logarithm of the waiting time distribution. We see that the latter, which gives more weight to the part of the waiting time distribution beyond the peak, provides a much better estimate for the interaction rate. We have therefore decided to extract the effective rates $\lambda(y)$ by least squares fitting of the logarithm of the experimental waiting time distributions (Fig. 2a, solid lines).

3. Experimental concentrations of *Chlamydomonas* cells across the experiments

Although we strived for a constant concentration of cells across all experiments, this cannot be controlled exactly. As a result, there were small differences in the measured 2D cell number concentration between different experiments. This has an effect on the rate of active encounters which, at these concentrations, is expected to be proportional to cell concentration [2]. The cell concentrations are given in Supplementary Table I.

Channel type	Channel Size μm	Cell Surface Density (cells/μm^2) ($\times 10^{-4}$)
Straight	50	2.7 ± 0.7
Straight	100	4.5 ± 0.8
Straight	150	2.9 ± 0.6
Straight	200	4.0 ± 0.7
Circular	200	3.8 ± 1.4

Supplementary Table I: Experimental cell density for the different experiments.

4. Derivation of the Fokker-Planck equation (Eq. 2)

Motivated by the numerical validation of the jump-diffusion dynamics, we aim to develop a general analytical model that accounts for the experimental results. Following Denisov and Bystrik [4] we first write a stochastic (Langevin)

equation for the colloidal dynamics:

$$Y_{t+\tau} = Y_t + N_\tau + \Delta_\tau(Y_t) \tag{1}$$

where τ is an infinitesimal time interval, Δ_τ is a random variable describing the inhomogeneous Poisson process and N_τ is a standard Wiener process. This can then be used to derive the Fokker-Planck equation (Eq. 2). We first write

$$\begin{aligned} P_{t+\tau}(y) &= \langle \delta(y - Y_{t+\tau}) \rangle, \\ &= \langle \delta(y - (Y_t + N_\tau + \Delta_\tau(Y_t)) \rangle, \end{aligned} \tag{2}$$

with the angular brackets denoting an average over all noise realizations, which we can express in the following way

$$P_{t+\tau}(y) = \int_{-\infty}^{+\infty} dy' P_t(y') \int_{-\infty}^{+\infty} \int_{-\infty}^{+\infty} dy_{\mathrm{J}}\, d\Delta\eta\, p_\tau^{y'}(y_{\mathrm{J}}) r_\tau(\Delta\eta) \delta(y - (y' + y_{\mathrm{J}} + \Delta\eta)), \tag{3}$$

where $p_\tau^{y'}(y_{\mathrm{J}})$ is the probability density that $\Delta_\tau = y_{\mathrm{J}}$ at position y' and $r_\tau(\Delta\eta) = e^{-\Delta\eta^2/(4D\tau)}/\sqrt{4\pi D\tau}$ is the transition probability for the Wiener process (with diffusion coefficient D) [5]. Integrating first over $\Delta\eta$ we get:

$$P_{t+\tau}(y) = \int_{-\infty}^{+\infty} dy' \frac{P_t(y')}{\sqrt{4\pi D\tau}} \int_{-\infty}^{+\infty} dy_{\mathrm{J}}\, p_\tau^{y'}(y_{\mathrm{J}}) e^{-\frac{(y-y'-y_{\mathrm{J}})^2}{4D\tau}}, \tag{4}$$

which we can Fourier-transform

$$\begin{aligned} \tilde{P}_k(t+\tau) &= \int_{-\infty}^{+\infty} dy' \frac{P_t(y')}{\sqrt{4\pi D\tau}} \int_{-\infty}^{+\infty} dy_{\mathrm{J}}\, p_\tau^{y'}(y_{\mathrm{J}}) \int_{-\infty}^{+\infty} dy\, e^{-iky} e^{-\frac{(y-y'-y_{\mathrm{J}})^2}{4D\tau}}, \\ &= \int_{-\infty}^{+\infty} dy' P_t(y') \int_{-\infty}^{+\infty} dy_{\mathrm{J}}\, p_\tau^{y'}(y_{\mathrm{J}}) e^{-ik(y'+y_{\mathrm{J}})} e^{-D\tau k^2}. \end{aligned} \tag{5}$$

Now we are going to expand Eq. 5 to first order in τ. Following [4], we can write

$$p_\tau^{y'}(y_{\mathrm{J}}) = (1 - \lambda(y')\tau)\delta(y_{\mathrm{J}}) + \lambda(y')\tau q_{y'}(y_{\mathrm{J}}) + o(\tau^2), \tag{6}$$

where $q_{y'}(y_{\mathrm{J}})$ is the probability of having a jump of size y_{J} at position y' and $\lambda(y')$ is the Poissonian rate at position y'. We can then get the first order expansion:

$$\begin{aligned} \tilde{P}_k(t+\tau) &\approx (1 - Dk^2\tau) \int_{-\infty}^{+\infty} dy'\, P_t(y') e^{-iky'} - \tau \int_{-\infty}^{+\infty} dy'\, P_t(y')\lambda(y') e^{-iky'} \\ &+ \tau \int_{-\infty}^{+\infty} \int_{-\infty}^{+\infty} dy_{\mathrm{J}}\, dy'\, P_t(y')\lambda(y') q_{y'}(y_{\mathrm{J}}) e^{-ik(y'+y_{\mathrm{J}})}, \\ &\approx (1 - Dk^2\tau)\tilde{P}_k(t) - \tau \int_{-\infty}^{+\infty} dy' P_t(y')\lambda(y') e^{-iky'} \\ &+ \tau \int_{-\infty}^{+\infty} \int_{-\infty}^{+\infty} dy_{\mathrm{J}} dy' P_t(y')\lambda(y') q_{y'}(y_{\mathrm{J}}) e^{-ik(y'+y_{\mathrm{J}})}. \end{aligned} \tag{7}$$

From this expansion, we can express the time-derivative of $\tilde{P}_k(t)$

$$\begin{aligned} \frac{\partial \tilde{P}_k(t)}{\partial t} &= \lim_{\tau\to 0} \frac{\tilde{P}_k(t+\tau) - \tilde{P}_k(t)}{\tau}, \\ &= -Dk^2 \tilde{P}_k(t) - \int_{-\infty}^{+\infty} dy' P_t(y')\lambda(y') e^{-iky'} \\ &+ \int_{-\infty}^{+\infty} \int_{-\infty}^{+\infty} dy_{\mathrm{J}}\, dy'\, P_t(y')\lambda(y') q_{y'}(y_{\mathrm{J}}) e^{-ik(y'+y_{\mathrm{J}})}. \end{aligned} \tag{8}$$

We can now inverse Fourier-transform Eq. 8 to get

$$\begin{aligned} \frac{\partial P_t(y)}{\partial t} &= D \frac{\partial^2 P_t(y)}{\partial y^2} - \int_{-\infty}^{+\infty} dy' P_t(y')\lambda(y')\delta(y - y') \\ &+ \int_{-\infty}^{+\infty} \int_{-\infty}^{+\infty} dy_{\mathrm{J}} dy' P_t(y')\lambda(y') q_{y'}(y_{\mathrm{J}}) \delta(y - y' - y_{\mathrm{J}}), \end{aligned} \tag{9}$$

and finally obtain Eq. 2

$$\frac{\partial P_t(y)}{\partial t} = D_0 \frac{\partial^2 P_t(y)}{\partial y^2} - \lambda(y) P_t(y) + \int_{-\infty}^{+\infty} \lambda(y - y_\mathrm{J}) P_t(y - y_\mathrm{J}) q_{y-y_\mathrm{J}}(y_\mathrm{J}) dy_\mathrm{J}. \tag{10}$$

5. Derivation of the drift-diffusion equation (Eq. 3): Kramers-Moyal expansion

From Eq. 10 above, we can perform a Kramers-Moyal expansion in order to get an effective drift-diffusion equation (Eq. 3). We have (rewriting $q_{y-y_\mathrm{J}}(y_\mathrm{J}) = q(y - y_\mathrm{J}, y_\mathrm{J})$)

$$\begin{aligned}\lambda(y - y_\mathrm{J}) P_t(y - y_\mathrm{J}) q(y - y_\mathrm{J}, y_\mathrm{J}) =& \lambda(y) P_t(y) q(y, y_\mathrm{J}) - y_\mathrm{J} \frac{\partial}{\partial y}\Big[\lambda(y) P_t(y) q(y, y_\mathrm{J})\Big] \\ &+ \frac{y_\mathrm{J}^2}{2} \frac{\partial^2}{\partial y^2}\Big[\lambda(y) P_t(y) q(y, y_\mathrm{J})\Big] + o(y_\mathrm{J}^3)\end{aligned} \tag{11}$$

which leads to

$$\begin{aligned}\frac{\partial P_t(y)}{\partial t} =& D \frac{\partial^2 P_t(y)}{\partial y^2} - \lambda(y) P_t(y) + \lambda(y) P_t(y) \int_{-\infty}^{+\infty} q_y(y_\mathrm{J}) dy_\mathrm{J} \\ &- \frac{\partial}{\partial y}\Big[\lambda(y) P_t(y) \int_{-\infty}^{+\infty} y_\mathrm{J} q_y(y_\mathrm{J}) dy_\mathrm{J}\Big] + \frac{\partial^2}{\partial y^2}\Big[\frac{\lambda(y) P_t(y)}{2} \int_{-\infty}^{+\infty} y_\mathrm{J}^2 q_y(y_\mathrm{J}) dy_\mathrm{J}\Big],\end{aligned} \tag{12}$$

where we recognize the moments of the distribution of jump size $q_y(y_\mathrm{J})$, $m_n(y) = \int_{-\infty}^{+\infty} y_\mathrm{J}^n q_y(y_\mathrm{J}) dy_\mathrm{J}$. Since $m_0(y) = 1$ for all y's, we have

$$\begin{aligned}\frac{\partial P_t(y)}{\partial t} =& \frac{\partial}{\partial y}\Big[\Big(D + \frac{\lambda(y) m_2(y)}{2}\Big) \frac{\partial P_t}{\partial y}\Big] \\ &- \frac{\partial}{\partial y}\Big[\Big(\lambda(y) m_1(y) - \frac{1}{2}\frac{\partial}{\partial y}[\lambda(y) m_2(y)]\Big) P_t(y)\Big]\end{aligned} \tag{13}$$

which is a drift-diffusion equation with effective diffusivity $D_\mathrm{eff}(y) = D + \frac{\lambda(y) m_2(y)}{2}$ and effective drift $V_\mathrm{eff}(y) = \lambda(y) m_1(y) - \frac{1}{2}\frac{\partial}{\partial y}[\lambda(y) m_2(y)]$.

6. Filling dynamics in the demixing experiments

In the demixing experiments, let us call $N_c(t)$ and $N_s(t)$ the total number of colloidal particles that at time t are in the circular chamber or in the side channels respectively. In a first-order kinetics, these obey the following linear evolution:

$$\frac{dN_c(t)}{dt} = -k_\mathrm{in} N_c(t) + k_\mathrm{out} N_s(t) \tag{14}$$

$$\frac{dN_s(t)}{dt} = +k_\mathrm{in} N_c(t) - k_\mathrm{out} N_s(t). \tag{15}$$

Notice that this assumes that the total number of colloids in the chamber and the side channels, $N_t = N_c + N_s$, is constant in time. This is what we observe in our experiments (purple solid line in Fig. 4b). This set of equations is immediately solved to give

$$N_c(t) = N_t \left[\frac{k_\mathrm{out} + k_\mathrm{in} e^{-(k_\mathrm{out} + k_\mathrm{in})t}}{k_\mathrm{out} + k_\mathrm{in}}\right] \tag{16}$$

$$N_s(t) = N_t \left[\frac{k_\mathrm{in}\left(1 - e^{-(k_\mathrm{out} + k_\mathrm{in})t}\right)}{k_\mathrm{out} + k_\mathrm{in}}\right]. \tag{17}$$

7. Estimating the rate of escape from a circular chamber into the side channels: k_{in}

We will outline two methods of differing complexity that can be used to predict the escape rate of the colloids $k_{\rm in}$, whose experimentally measured value is $k_{\rm in} = (7.8 \pm 0.6) \times 10^{-5}\,{\rm s}^{-1}$. The system we consider is composed of a single particle within a circular chamber, subject to diffusion and -later- drift. The walls of the circular chamber divided into two types: the first is a no-flux part which the colloids cannot penetrate; the second is an absorbing part where the colloids are removed from the system. We will estimate the escape rate as the inverse of the average time taken by a colloid to be absorbed at the boundary. This is of course a version of the famous 'Narrow Escape Problem' [6] with two variations. Firstly, the particles are subject to a space-dependent diffusivity and drift, while the Narrow Escape approaches generally have constant diffusivity and no drift. Secondly, in order to stay faithful to the geometry of the experiments, the boundary is composed of several distinct absorbing patches, rather than a single one (of the same total size) as would be standard in the Narrow Escape Problem.

For a single absorbing patch at the boundary of a disk, the Narrow Escape Problem has been solved for a particle with constant diffusivity in [7]. Following this work we can begin noting that solving the mean first passage time, and hence escape rate, means solving the following Poisson equation with mixed Neumann-Dirichlet inhomogeneous boundary conditions:

$$\begin{cases} D\Delta t(r,\theta) = -1 & \text{for } r < R,\ 0 \le \theta < 2\pi \\ t(r,\theta) = 0 & \text{for } r = R,\ \theta \in \Theta_a \\ \frac{\partial t(r,\theta)}{\partial r} = 0 & \text{for } r = R,\ \theta \notin \Theta_a \end{cases} \tag{18}$$

where (r,θ) are the coordinates on the disk of radius R, D the constant diffusivity, $t(r,\theta)$ the escape time given initial position (r,θ) and Θ_a is the set of angles for which the boundary is absorbing. In this case this is a set of 12 regions with angles corresponding to 7.5μm exits. This set of equations can then be solved numerically for a given prescribed boundary and diffusivity to give the escape rates of the colloids.

D (μm^{2}s^{-1})	$t(0,0)^{-1}$ ($\times 10^{-5}$ s^{-1})	$\langle t(r,\theta)\rangle^{-1}$ ($\times 10^{-5}$ s^{-1})	$\langle t(r,\theta)\rangle_\rho^{-1}$ ($\times 10^{-5}$ s^{-1})
$D_{\rm th} = 0.05$	0.228	0.295	0.280
$D_{\rm bulk} = 3.55$	16.2	20.8	19.9
$\langle D(r,\theta)\rangle = 3.14$	14.3	18.3	17.6
$\langle D(r,\theta)\rangle_\rho = 3.12$	14.2	18.3	n/a

Supplementary Table II: Escape rates for constant diffusivities and no drift.

Supplementary Table II shows the rates obtained from the numerical solution of Eq. (18) for four different values for the constant diffusivity: i) the bulk thermal value (to be used as a baseline); ii) the bulk effective diffusivity $D_{\rm bulk}$ predicted by the Kramers-Moyal (KM) model; iii) the spatially-averaged KM diffusivity over the whole system, $\langle D(r,\theta)\rangle$; iv) the average KM diffusivity weighted by the stationary colloidal distribution ρ, $\langle D(r,\theta)\rangle_\rho$. For each fixed diffusivity, we report the escape rates calculated with i) a fixed starting point at the centre of the chamber ($t(0,0)^{-1}$); ii) a uniformly distributed initial particle position ($\langle t(r,\theta)\rangle^{-1}$); iii) an initial particle position distributed according to ρ ($\langle t(r,\theta)\rangle_\rho^{-1}$). It is clear that all these rates largely overestimate the experimental one.

Up to this point we have limited ourselves to a constant diffusivity and no particle drift, but we have seen in the KM model that such features are required to recapitulate the colloidal distributions. In order to include them in the estimate of the escape, we perform a numerical simulation of a colloid subject to the space-dependent effective diffusivity and drift used in the KM model. We expect this to be a minimal model for the dynamics of individual colloids, for timescales long compared to the waiting time between successive jumps. To simulate the discrete dynamics, we adapt to our case the so-called Milstein method:

$$\begin{aligned} x(t+\delta t) &= x(t) + \sqrt{2D(r)\delta t}\,\xi_1(t) + v_1(r)\delta t\cos(\theta) + v_2(r)\delta t\cos(\theta), \\ y(t+\delta t) &= y(t) + \sqrt{2D(r)\delta t}\,\xi_2(t) + v_1(r)\delta t\sin(\theta) + v_2(r)\delta t\sin(\theta), \end{aligned} \tag{19}$$

Here, $(x(t), y(t))$ is the position of the colloid at time t, $D(r) = D_0 + \frac{1}{2}\lambda(r)m_2(r)$ is the local effective diffusivity at a position $r = |(x,y)|$, $\xi(\cdot)$ is a Gaussian white noise of variance 1, $v_1(r) = \lambda(r)m_1(r)$ is the drift due to the first moment of the jump distributions, $v_2(r) = \frac{1}{2}D'(r)\left(\xi_i(t)^2 - \alpha\right)$ the drift due to the second moment. The constant α captures the integration scheme [8], with $\alpha = 0, 1$ corresponding to the Stratonovitch and Itô respectively. The angle

$\theta = \arctan(y(t)/x(t))$ ensures the drifts are oriented towards the center of the chamber. Of course, simulations allow the boundary conditions to match those in the experiment.

In our simulations we use: $\delta t = 0.1$s; the same boundary structure used to estimate the values of Supplementary Table II; and the KM effective diffusivity and drift calculated for the 100 μm channel rescaled by the ratio of the concentrations between that experiment and the circular chamber one. The results can be found in Supplementary Table III for the escape rate given an initial condition of particles at the centre of the chamber, where the error is the stochastic error from the simulations.

α	$t(0,0)^{-1}$ ($\times 10^{-5}$ s^{-1})	$\pm$ stochastic error from simulations ($\times 10^{-5}$ s^{-1})
0 (Stratonovitch)	7.408	0.066
1 (Ito)	3.063	0.027

Supplementary Table III: Escape rates calculated from Eq. 19.

SUPPLEMENTARY FIGURES

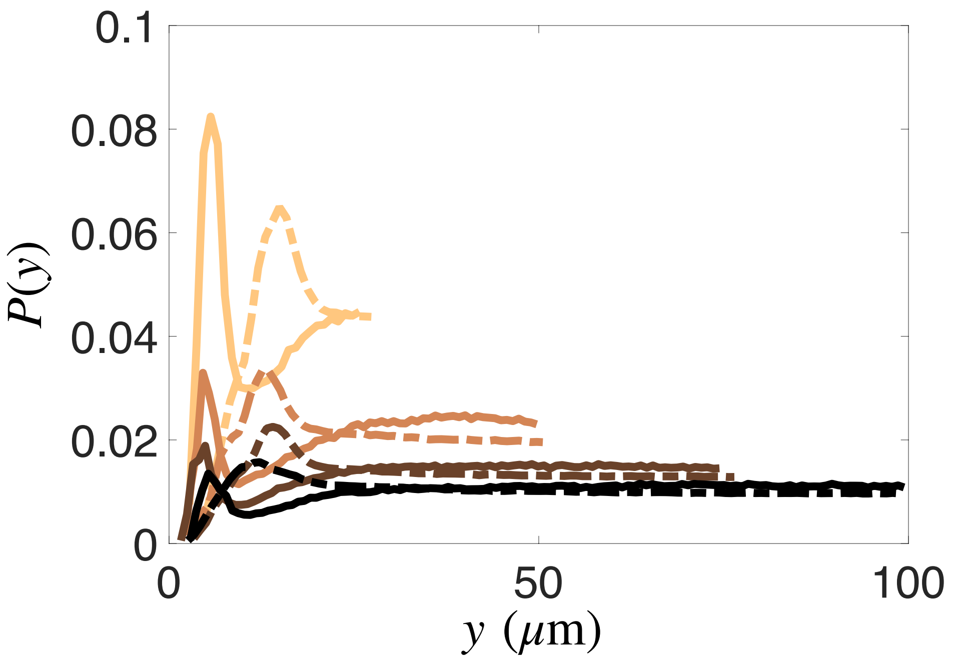

Supplementary Figure 1: Steady state distribution function of algae (dash-dotted lines) and colloids (solid lines, same data as in Fig. 1b) across the straight channels, from the boundary to the channel midpoint. The algal distribution displays a clear peak at $y_{\mathrm{CR}} \approx 15\ \mu\mathrm{m}$.

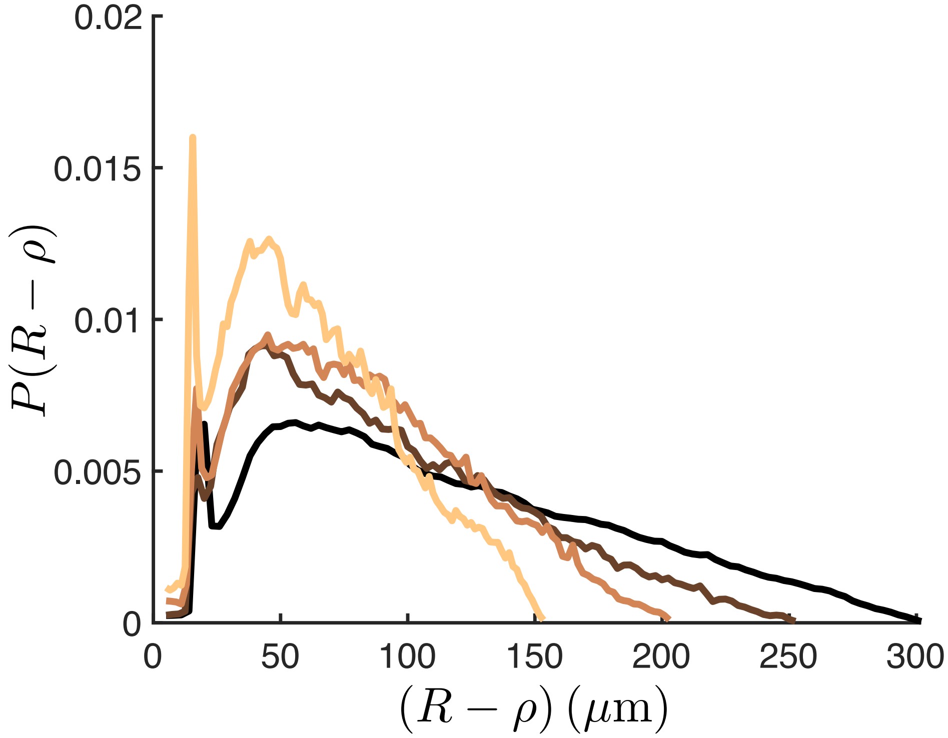

Supplementary Figure 2: Steady state probability distribution function $P(\rho)$ for colloids in active-passive experiments, within circular chambers of radius R. By symmetry, the probability distribution function is only a function of the radial coordinate ρ. The probability distribution function is defined in such a way that $\int_0^R P(\rho)\mathrm{d}\rho = 1$.

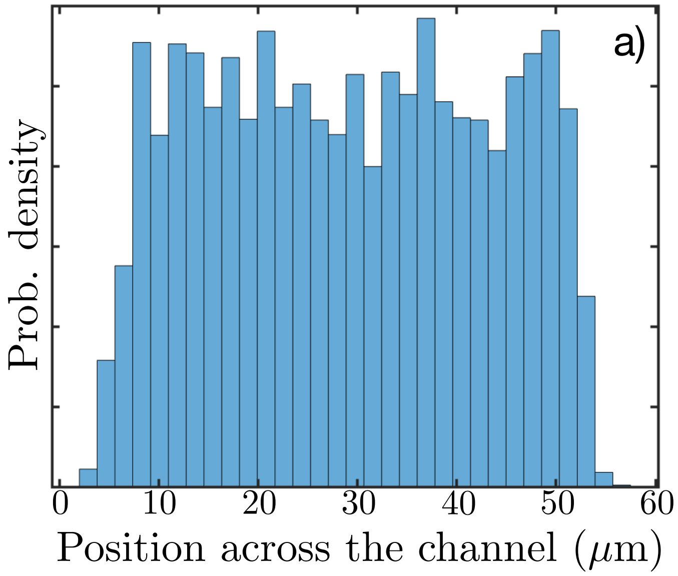

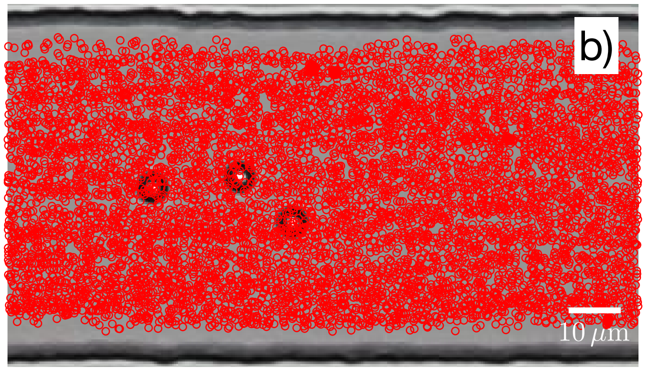

Supplementary Figure 3: Steady state colloidal distribution, control case. a) Steady-state distribution of 6 μm-diameter PS-COOH colloids within a microfluidic channel 57.5 μm wide and 14.5 μm-thick. b) Same data as in the previous panel, visualised as actual colloidal positions along the channel.

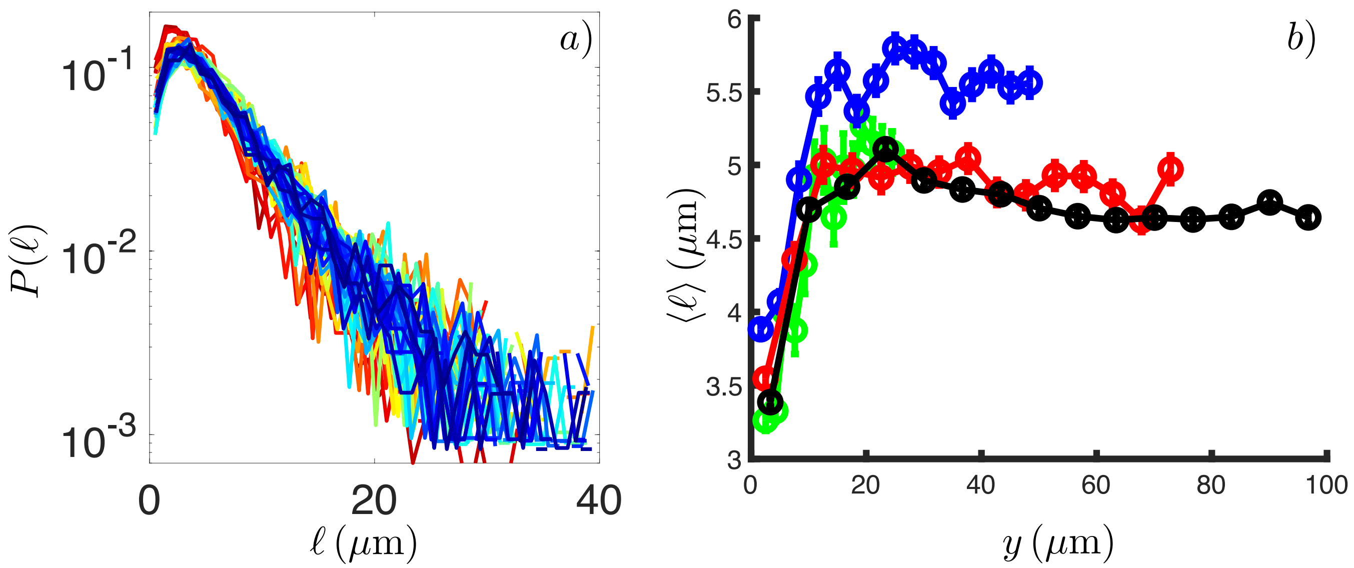

Supplementary Figure 4: a) Probability distribution functions of jump lengths $P(l)$ at different distances y from the channel boundary (in the $2W = 100\mu$m channel). Semi-log plot. The colour code is the same as in Fig. 2 of the main manuscript (red at the boundary, blue in the middle of the channel). b) Position-dependent average jump magnitude $\langle \ell \rangle (y)$ for the four values of W explored ($2W = 50, 100, 150, 200\,\mu$m). Error bars from fit uncertainties.

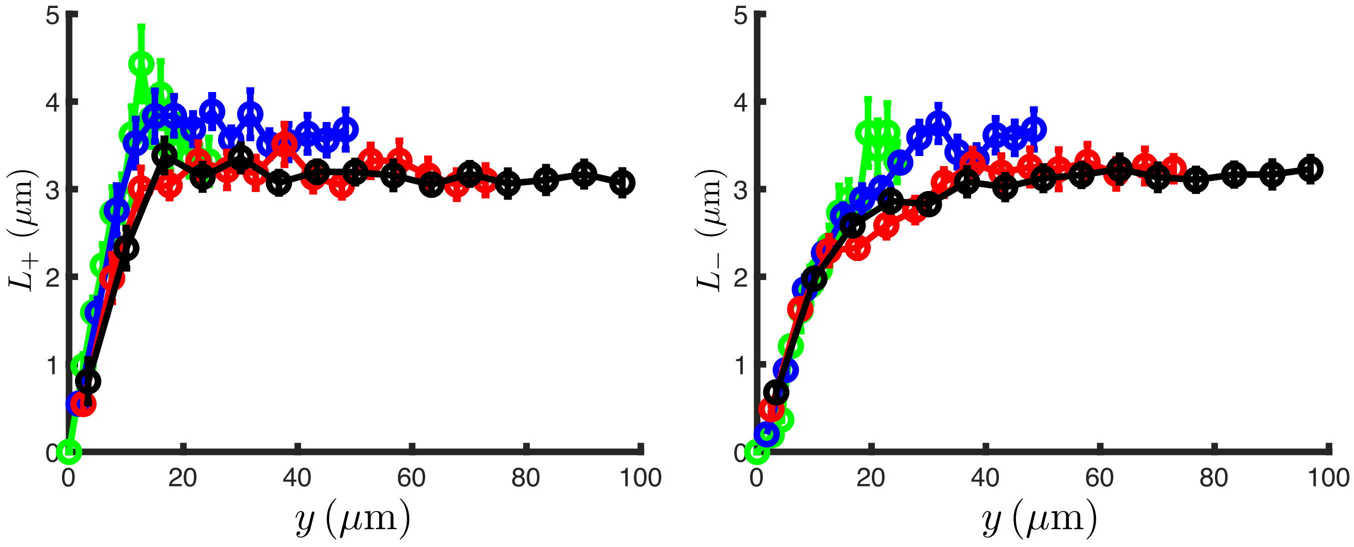

Supplementary Figure 5: The characteristic lengths L_+ and L_- as a function of distance y from the nearest boundary. The colours (green, blue, red, black) correspond to the four values of W explored ($2W = 50, 100, 150, 200\,\mu$m). Error bars from fit uncertainties.

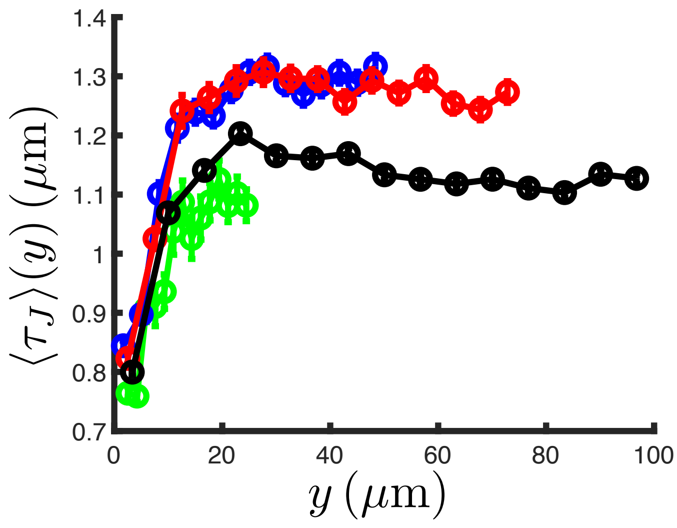

Supplementary Figure 6: Average duration of a jump event $\langle \tau_J \rangle$(y) as a function of distance y from the nearest boundary. The colours (green, blue, red, black) correspond to the four values of W explored ($2W = 50, 100, 150, 200\,\mu$m). Error bars are standard errors of the mean.

SUPPLEMENTARY REFERENCES

[1] L. S. Mosby, M. Polin, and D. V. Köster, *A Python based automated tracking routine for myosin II filaments*, J. Phys. D: Appl. Phys **53**, 304002 (2020).

[2] R. Jeanneret, D. O. Pushkin, V. Kantsler, and M. Polin, *Entrainment dominates the interaction of microalgae with micron-sized objects*, Nat. Commun. **7**, 12518 (2016).

[3] K. C. Leptos, J. S. Guasto, J. P. Gollub, A. Pesci, and R. E. Goldstein, *Dynamics of Enhanced Tracer Diffusion in Suspensions of Swimming Eukaryotic Microorganisms*, Phys. Rev. Lett. **103**, 198103 (2009).

[4] S. I. Denisov and Y. S. Bystrik, *Statistics of bounded processes driven by Poisson white noise*, Physica A **515**, 38 (2019).

[5] S. I. Denisov, W. Horsthemke, and P. Hänggi, *Generalized Fokker-Planck equation: Derivation and exact solutions*, European Physical Journal B **68**, 567 (2009).

[6] S. Redner, *A Guide to First-Passage Processes* (Cambridge University Press, Cambridge, 2001).

[7] A. Singer, Z. Schuss, D. Holcman, and R. S. Eisenberg, *Narrow escape, part I*, Journal of Statistical Physics **122**, 437 (2006).

[8] P. Lançon, G. Batrouni, L. Lobry, and N. Ostrowsky, *Drift without flux: Brownian walker with a space-dependent diffusion coefficient*, Europhys. Lett. **54**, 28 (2001), 0005004.
