# Supplementary material for: Confinement-induced accumulation and de-mixing of microscopic active-passive mixtures
Source: Nat Commun. 2022 Aug 15;13:4776. doi: 10.1038/s41467-022-32520-9 (PMC9378696; doi:10.1038/s41467-022-32520-9)
Supplement: Supplementary file 3 — Description of Additional Supplementary Files [file 41467_2022_32520_MOESM3_ESM.pdf]

Description of Additional Supplementary Files

Supplementary Movie 1: C. reinhardtii microalgae and 10μm-diameter polystyrene colloids within a set of microfluidic straight channels. Recorded at 10 frames per second; length conversion factor 0.55 μm per pixel.

Supplementary Movie 2: Colloid-alga interaction showing a single colloid with its trajectory indicated as it interacts with several microoganisms. Recorded at 10 frames per second; length conversion factor 0.55 μm per pixel.

Supplementary Movie 3: Colloid-alga interaction influencing the subsequent swimming of the microorganism and causing it rapid successive interactions. Recorded at 10 frames per second; length conversion factor 0.55 μm per pixel.

Supplementary Movie 4: Dynamics of side channels filling with colloids due to activity of the microalgae. Recorded at 0.1 frames per second; length conversion factor 0.23 μm per pixel.

Supplementary Movie 5: Same as Supplementary Movie 4 but without the microalgae in the main chamber. Recorded at 0.25 frames per second; length conversion factor 0.55 μm per pixel.
